# Protective effect of baicalein from *Pinellia ternate* on Alzheimer’s disease cell injury: a network pharmacology, molecular docking, and molecular dynamics study

**DOI:** 10.3389/fnagi.2026.1848282

**Published:** 2026-06-11

**Authors:** Tuo Ji, Lin Wang, Xu Weng, Chang Lu, Yuzhi Gao, Kun Yu, Jiachen He, Xiaozhu Shen, Xuzhu Gao

**Affiliations:** 1 The Second People’s Hospital of Lianyungang Affiliated to Kangda College of Nanjing Medical University, Lianyungang, China; 2 Lianyungang Clinical College, Bengbu Medical University, Lianyungang, China; 3 Department of Central Laboratory, The Second People’s Hospital of Lianyungang, Lianyungang, China; 4 Department of Geriatrics, The Second People’s Hospital of Lianyungang, Lianyungang, China

**Keywords:** Alzheimer’s disease, *Pinellia ternata*, network pharmacology, molecular docking, molecular dynamics simulation, baicalein

## Abstract

**Introduction:**

Alzheimer’s disease (AD) constitutes the primary leading cause of dementia. Pinellia ternata (Thunb.) Breit. is a traditional Chinese herb with unclarified potential therapeutic effects against AD. This study aimed to explore the therapeutic potential and underlying mechanism of *Pinellia ternata* (Thunb.) Breit. in the treatment of AD.

**Methods:**

The bioactive components and corresponding targets of *Pinellia ternata* (Thunb.) Breit. were screened from TCMSP, Herb, and SymMap databases. AD-related targets were retrieved from OMIM, GeneCards, and TTD databases, and key targets were obtained via target intersection analysis. Functional enrichment analyses were performed to identify the main signaling pathways involved in the targets. Core targets and major bioactive components were further screened, and molecular docking as well as dynamics simulations were conducted to verify the binding affinity between key components and core targets. In vitro cell experiments using BV2 cells were implemented to validate the therapeutic effect of the core bioactive component.

**Results:**

A total of 13 bioactive components and 99 corresponding targets of *Pinellia ternata* (Thunb.) Breit. were identified, and 29 key AD-related targets were screened out through target intersection. Enrichment analysis results showed that these key targets were mainly enriched in neuroactive ligand-receptor interaction and calcium signaling pathways. PTGS2, CASP2, and AKT1 were determined as core therapeutic targets, with β-sitosterol and baicalein identified as the principal bioactive components of *Pinellia ternata* (Thunb.) Breit. against AD. Molecular docking and dynamics simulations verified the strong binding affinity between baicalein and PTGS2. *In vitro* experimental results further demonstrated that baicalein pretreatment could relieve the inhibitory effect of Aβ_1-42_ on BV2 cell proliferation.

**Discussion:**

*Pinellia ternata* (Thunb.) Breit. exerts therapeutic effects on AD via a synergistic mechanism characterized by multi-component, multi-target, and multi-pathway regulation. The active ingredient baicalein targeting PTGS2 is a crucial material basis for its anti-AD effect. The findings of this study elucidate the potential mechanism of *Pinellia ternata* (Thunb.) Breit. in AD treatment and provide a reliable theoretical foundation for subsequent in-depth research and clinical exploration of the herb as a therapeutic agent for AD.

## 1 Introduction

Alzheimer’s disease (AD) is the leading cause of dementia, with its prevalence rising annually in parallel with global population (Hodson, 2018; Zheng and Wang, 2025). In 2018, the International Alzheimer’s Disease Association estimated that approximately 50 million individuals worldwide were living with AD. The number is projected to triple by 2050, with the vast majority of cases occurring in developing countries (Scheltens et al., 2021). The pathological hallmarks of AD include the accumulation of amyloid plaques and neurofibrillary tangles in the brain, ultimately leading to neurodegeneration and cognitive decline (Joe and Ringman, 2019; Lee et al., 2024). In addition to genetic predisposition, the onset and progression of AD are influenced by multiple risk factors, including aging, systemic inflammation, lifestyle, and environmental exposures (Zheng and Wang, 2025). Currently, the U. S. Food and Drug Administration (FDA) has approved several pharmacological interventions for AD, including cholinesterase inhibitors, N-methyl-D-aspartate (NMDA) receptor antagonists, and Aβ-targeting monoclonal antibodies. These treatments primarily aim at symptom management or modulation of specific pathological pathways, yet they are unable to reverse or halt disease progression. Moreover, their clinical utility is limited by notable adverse effects and high treatment costs (Dhillon, 2021; Vaz et al., 2022). Consequently, the exploration of novel therapeutic strategies capable of targeting the complex and multifactorial mechanisms of AD in a multi-target manner—particularly through the investigation of promising natural products derived from traditional Chinese medicine (TCM)—has emerged as a critical research direction.

TCM has accumulated millennia of empirical experience in the prevention and treatment of chronic, complex diseases. Its theoretical framework, characterized by a holistic perspective and syndrome differentiation-guided therapy, combined with system-level regulation strategy targeting multiple biological pathways, provides a unique therapeutic approach for multifactorial diseases (Wang et al., 2015). *Pinellia ternata* (Thunb.) Breit. is the dried tuber of Araceae. It is pungent in taste, warm in nature, toxic, and associated with the spleen, stomach, and lung meridians. Pharmacological studies have shown that *Pinellia ternata* (Thunb.) Breit. exhibits activities including drying dampness and resolving phlegm, counteracting adverse qi and preventing vomiting, dispersing masses and resolving nodules, as well as anti-tumor, antibacterial, anti-inflammatory, and antiepileptic effects (Xu et al., 2018).

Emerging evidence suggests that *Pinellia ternata* (Thunb.) Breit. may hold therapeutic potential for neurological disorders. However, its efficacy in AD, the active components responsible, the multi-target action network, and the underlying molecular mechanisms remain largely unexplored.

The concept of network pharmacology has emerged as a novel analytical methodology and research paradigm in recent years, integrating principles from systems biology, medical science, and big data analysis. It facilitates the systematic mapping of interactions between bioactive drug components and their molecular targets through visual networks (Wang et al., 2024). Network pharmacology particularly well-aligned with the foundational principles of TCM, it adopts a “multi-component, multi-target” framework to predict the mechanisms of action of herbal medicines. Molecular docking and molecular dynamics (MD) simulations are key computational experimental techniques that model the binding modes and affinities between small-molecule ligands and biological macromolecular targets at atomic resolution, providing essential validation for predictions generated through network pharmacology. The integration of these three approaches establishes an efficient and feasible framework for elucidating the complex mechanisms underlying the pharmacological effects of TCM.

In this study, a network pharmacology approach was employed to systematically identify the active components of *Pinellia ternata* (Thunb.) Breit., and predict its potential therapeutic targets for AD. A multi-dimensional component-target-pathway network was constructed to provide a holistic view of its pharmacological interactions. Gene Ontology (GO) functional enrichment analysis and Kyoto Encyclopedia of Genes and Genomes (KEGG) pathway enrichment analysis were conducted to comprehensively characterize the potential mechanisms of action. Additionally, molecular docking and MD simulations were performed to validate interactions between the core active components and key targets, and to assess the stability of these interactions. Finally, BV2 microglial cells were selected as the initial experimental model, with subsequent studies planned to further evaluate efficacy in neuronal and co-culture systems.

## 2 Methods

### 2.1 Analysis of main active components and targets of *Pinellia ternata* (Thunb.) Breit

The active components of *Pinellia ternata* (Thunb.) Breit. were retrieved from the TCMSP database, the Herb database and the SymMap database. Potential active components were screened using thresholds of oral bioavailability (OB) ≥ 30% and drug-likeness (DL) ≥ 0.18 (Gao et al., 2024; Liu et al., 2025). In addition, metabolic metabolites of *Pinellia ternata* (Thunb.) Breit. components following oral administration were summarized from relevant literature and incorporated into the candidate component set for subsequent target prediction. Target prediction for the identified active components was performed via the TCMSP database, and protein target information was standardized using the UniProt database.

### 2.2 Analysis of AD targets

AD-related target genes were obtained from the OMIM, GeneCards, and Therapeutic Target Database (TTD) using “Alzheimer’s disease” as the keyword. Targets from each database were merged, and duplicate genes were removed to generate a comprehensive AD-related target set.

### 2.3 Protein–protein interaction (PPI) of active components of *Pinellia ternata* (Thunb.) Breit. and AD targets

To elucidate interactions between the targets of the active components of *Pinellia ternata* (Thunb.) Breit. and AD-associated targets, overlapping genes were identified as key drug action targets, visualized via a Venn diagram using the R language VennDiagram package. A PPI network model was subsequently constructed using the STRING database. The resulting network was imported into Cytoscape software, and the MCODE plugin was applied to identify and analyze functional protein modules.

### 2.4 Pathway enrichment analysis of active components of *Pinellia ternata* (Thunb.) Breit. and AD targets

Functional characterization and pathway involvement of the key targets were explored through enrichment analyses performed using the DAVID database, including GO enrichment and KEGG pathway enrichment analysis. GO analysis was conducted across three categories: Biological Process (BP), Cellular Component (CC), and Molecular Function (MF).

### 2.5 Molecular docking validation

Core targets with a degree greater than 10 in the PPI network were selected for molecular docking validation with key bioactive compounds. Three-dimensional (3D) structures of the core targets were downloaded from the UniProt database, imported into PyMOL software for ligand separation, and subsequently processed in AutoDockTools, which included dehydration, addition of hydrogen atoms, and receptor definition. The key compounds of *Pinellia ternata* (Thunb.) Breit. were downloaded in mol2 format from the TCMSP database, imported into AutoDockTools, hydrogenated, and defined as ligands. Molecular docking was performed using AutoDock Vina (Trott and Olson, 2010), and the optimal binding conformations were selected and visualized using PyMOL.

### 2.6 Molecular dynamics simulation

MD simulations were performed using GROMACS 2022.2. The protein was modeled with the Amber14SB force field, and the solvent was represented by the TIP3P water model. The small molecules were parameterized using antechamber to generate AM1-BCC charges and assign GAFF2 atom types, followed by conversion to GROMACS topology format via ACPYPE. Ion parameters compatible with TIP3P (Joung-Cheatham) were applied. The complex was placed in a truncated octahedral box with a minimum distance of ≥1.2 nm between the protein surface and the box boundary. TIP3P water molecules were added, and Na^+^/Cl^−^ ions were supplemented to neutralize the system and achieve a final concentration of 0.15 M. The system underwent energy minimization using the steepest descent method until the maximum force (Fmax) was <1,000 kJ·mol^−1^·nm^−1^. This was followed by a two-step equilibration process: first, a 200 ps NVT equilibration at 298 K, and then a 200 ps NPT equilibration at 298 K and 1 bar. Production MD simulations were performed under NPT conditions for 100 ns with a time step of 2 fs, employing the Verlet cutoff scheme. Coulomb interactions were treated using the Particle Mesh Ewald (PME) method. Van der Waals and Coulomb cutoff distances were both set to 1.2 nm. All bonds involving hydrogen atoms were constrained using the LINCS algorithm. Temperature and pressure were maintained at 298 K and 1 bar using the Nose-Hoover and Parrinello-Rahman, respectively. Trajectories were saved every 10 ps. Post-simulation analyses, including structural and interaction analyses, were performed using GROMACS built-in tools, VMD, and PyMOL. Binding free energies were evaluated using the gmx_MMPBSA tool.

### 2.7 Bioinformatics analysis

The gene expression dataset GSE97760 was obtained from the Gene Expression Omnibus (GEO) database. This dataset includes sequencing data from 9 blood samples of AD patients and 10 control samples. Quality control and normalization of the microarray data were performed using GEO2R. Subsequently, the data were standardized, and differentially expressed genes (DEGs) were identified using thresholds of adjusted *p*-value < 0.05 and absolute log_2_ fold change (|log_2_FC|) > 1.

### 2.8 Cell culture and viability assay

BV2 cells were procured from Shanghai Fuheng Biotechnology Co., Ltd. (China) and cultured in high-glucose DMEM medium supplemented with 10% fetal bovine serum at 37 °C with 5% CO_2_. Baicalein (purity ≥ 98%) was purchased from Yuanye Biotechnology Co., Ltd. (Shanghai, China). The reference standard was stored at 4 °C and prepared with DMEM. For the cell viability assay, BV2 cells were seeded into 96-well plates at a density of 30,000 cells per well and cultured overnight. In the model group, after removal of the original medium, cells were incubated with 10 μM or 20 μM Aβ_1-42_ (Qiangyao Biology) for 24 h to establish an AD model (Liu et al., 2026). In the treatment group, BV2 cells were pretreated with baicalein for 2 h (Yan et al., 2020; Gao et al., 2023). Following removal of the drug-containing medium, cells were further incubated with Aβ_1-42_ for 24 h. After treatment, the medium was replaced with fresh medium containing 10% CCK8 reagent, absorbance at 450 nm was measured after 2 h of incubation at 37 °C using a microplate reader. mRNA levels of PTGS2, AKT1, PGE2, NFKB1, TNFα, and IL6 were quantified by qPCR to evaluate inflammatory pathway activation.

### 2.9 Cell cycle and apoptosis analysis by flow cytometry

Cells in the exponential growth phase were seeded into 6-well plates and treated according to the respective experimental groups. After treatment, cells were digested using trypsin without EDTA, washed twice with PBS, and subsequently analyzed by flow cytometry using a cell cycle detection kit and an Annexin V-FITC/PI apoptosis detection kit.

### 2.10 Statistical analysis

Data are presented as mean ± standard error of the mean (SEM). Comparisons between two groups were performed using Student’s *t*-test, while differences among multiple groups were analyzed by one-way analysis of variance (ANOVA). A *p* < 0.05 was considered statistically significant. Graphs were prepared using GraphPad Prism. All experiments were performed in at least three independent replicates.

## 3 Results

### 3.1 Acquisition of active components of *Pinellia ternata* (Thunb.) Breit. and disease targets

A total of 13 active components of *Pinellia ternata* (Thunb.) Breit. were identified using the TCMSP, Herb, and SymMap databases, as well as supplementary data from previous studies (Table 1), corresponding to 99 predicted targets. Meanwhile, AD-related target genes were retrieved and integrated from the OMIM, GeneCards, and TTD databases. After removal of duplicate entries, 839 AD-associated targets were obtained.

**Table 1:** Active components of *Pinellia ternata* (Thunb.) Breit.

MOL ID	Molecule name	OB%	DL
MOL001755	24-Ethylcholest-4-en-3-one	36.08	0.76
MOL002670	Cavidine	35.64	0.81
MOL002714	baicalein	33.52	0.21
MOL002776	Baicalin	40.12	0.75
MOL000358	beta-sitosterol	36.91	0.75
MOL000449	Stigmasterol	43.83	0.76
MOL005030	gondoic acid	30.7	0.2
MOL000519	coniferin	31.11	0.32
MOL006936	10,13-eicosadienoic	39.99	0.2
MOL006937	12,13-epoxy-9-hydroxynonadeca-7,10-dienoic acid	42.15	0.24
MOL006957	(3S,6S)-3-(benzyl)-6-(4-hydroxybenzyl) piperazine-2,5-quinone	46.89	0.27
MOL003578	Cycloartenol	38.69	0.78
MOL006967	beta-D-Ribofuranoside, xanthine-9	44.72	0.21

### 3.2 Functional and pathway enrichment analysis of targets

**Figure 1:**
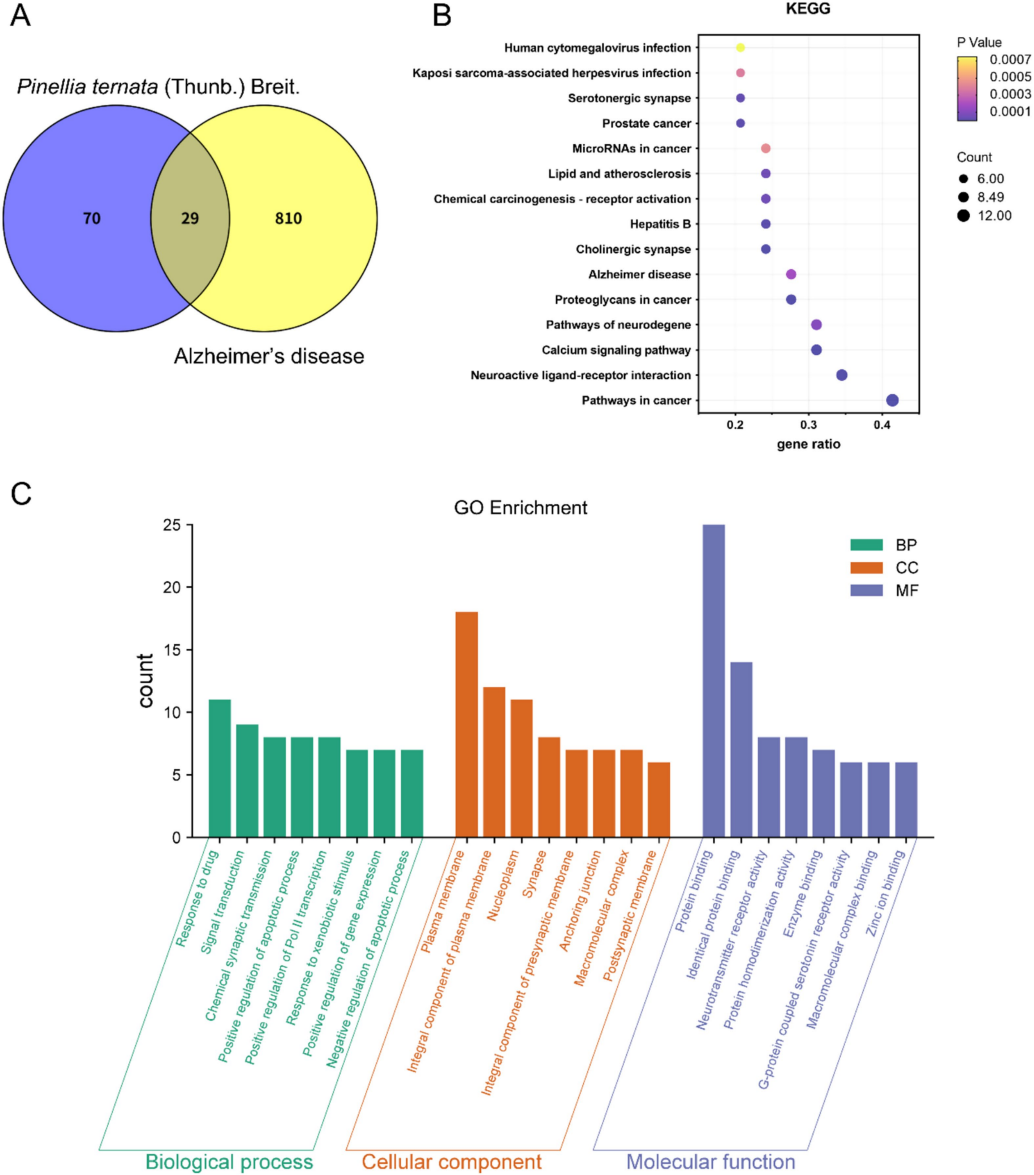
Gene enrichment analysis of genes associated with *Pinellia ternata* (Thunb.) Breit. and AD. **(A)** Venn diagram illustrating the overlap between the target genes of *Pinellia ternata* (Thunb.) Breit. and AD-related genes. **(B)** KEGG pathway analysis of target genes. **(C)** GO enrichment analysis of target genes.

The intersection between the 99 predicted targets of *Pinellia ternata* (Thunb.) Breit. and the 839 AD-related targets were determined, and a Venn diagram was generated using R, revealing 29 overlapping core targets (Figure 1A). Functional enrichment analysis of these 29 shared targets was performed using the DAVID database for both KEGG and GO analyses. The results were visualized using GraphPad Prism (Figures 1B,C). KEGG pathway enrichment analysis indicated that the signaling pathways associated with *Pinellia ternata* (Thunb.) Breit. mainly included Pathways in cancer, Neuroactive ligand-receptor interaction, Calcium signaling pathway, and Pathways of neurodegeneration, among others (Table 2). GO enrichment analysis showed that *Pinellia ternata* (Thunb.) Breit. is primarily involved in biological processes such as drug response, signal transduction, chemical synaptic transmission, positive regulation of apoptotic processes, and positive regulation of transcription from RNA polymerase II promoter. Cellular component analysis revealed enrichment in the plasma membrane, nucleoplasm, and synapse. Molecular function analysis indicated enrichment in protein binding, neurotransmitter receptor activity, protein homodimerization activity, and G-protein coupled serotonin receptor activity.

**Table 2:** KEGG enrichment.

Term	Description	Count	-Log10(P)	Genes
hsa05200	Pathways in cancer	12	6.40	AR, TGFB1, CASP8, CASP3, BCL2, AKT1, PPARG, PTGS2, ESR1, TP53, MMP9, VEGFA
hsa04080	Neuroactive ligand-receptor interaction	10	5.86	CHRM2, CHRM3, CHRM1, GABRA5, CHRNA7, CHRM5, HTR2C, ADRB2, HTR2A, ADRA2C
hsa04020	Calcium signaling pathway	9	6.16	CHRM2, CHRM3, CHRM1, CHRNA7, CHRM5, HTR2C, ADRB2, HTR2A, VEGFA
hsa05022	Pathways of neurodegeneration - multiple diseases	9	3.98	CHRM3, CHRM1, CASP8, CHRNA7, CASP3, CHRM5, BCL2, PTGS2, SLC6A3
hsa05205	Proteoglycans in cancer	8	5.48	TGFB1, PLAU, CASP3, AKT1, ESR1, TP53, MMP9, VEGFA
hsa05010	Alzheimer disease	8	3.72	CHRM3, CHRM1, CASP8, CHRNA7, CASP3, CHRM5, AKT1, PTGS2
hsa04725	Cholinergic synapse	7	5.84	CHRM2, CHRM3, CHRM1, CHRNA7, CHRM5, BCL2, AKT1
hsa05161	Hepatitis B	7	4.93	TGFB1, CASP8, CASP3, BCL2, AKT1, TP53, MMP9
hsa05207	Chemical carcinogenesis - receptor activation	7	4.27	AR, CHRNA7, BCL2, AKT1, ADRB2, ESR1, VEGFA
hsa05417	Lipid and atherosclerosis	7	4.23	CASP8, CASP3, BCL2, AKT1, PPARG, TP53, MMP9
hsa05206	MicroRNAs in cancer	7	3.36	PLAU, CASP3, BCL2, PTGS2, TP53, MMP9, VEGFA
hsa05215	Prostate cancer	6	4.85	AR, PLAU, BCL2, AKT1, TP53, MMP9
hsa04726	Serotonergic synapse	6	4.49	MAOB, CASP3, HTR2C, HTR2A, PTGS2, SLC6A4
hsa05167	Kaposi sarcoma-associated herpesvirus infection	6	3.42	CASP8, CASP3, AKT1, PTGS2, TP53, VEGFA
hsa05163	Human cytomegalovirus infection	6	3.12	CASP8, CASP3, AKT1, PTGS2, TP53, VEGFA

### 3.3 Construction of the active components of *Pinellia ternata* (Thunb.) Breit.-AD targets-pathways network

**Figure 2:**
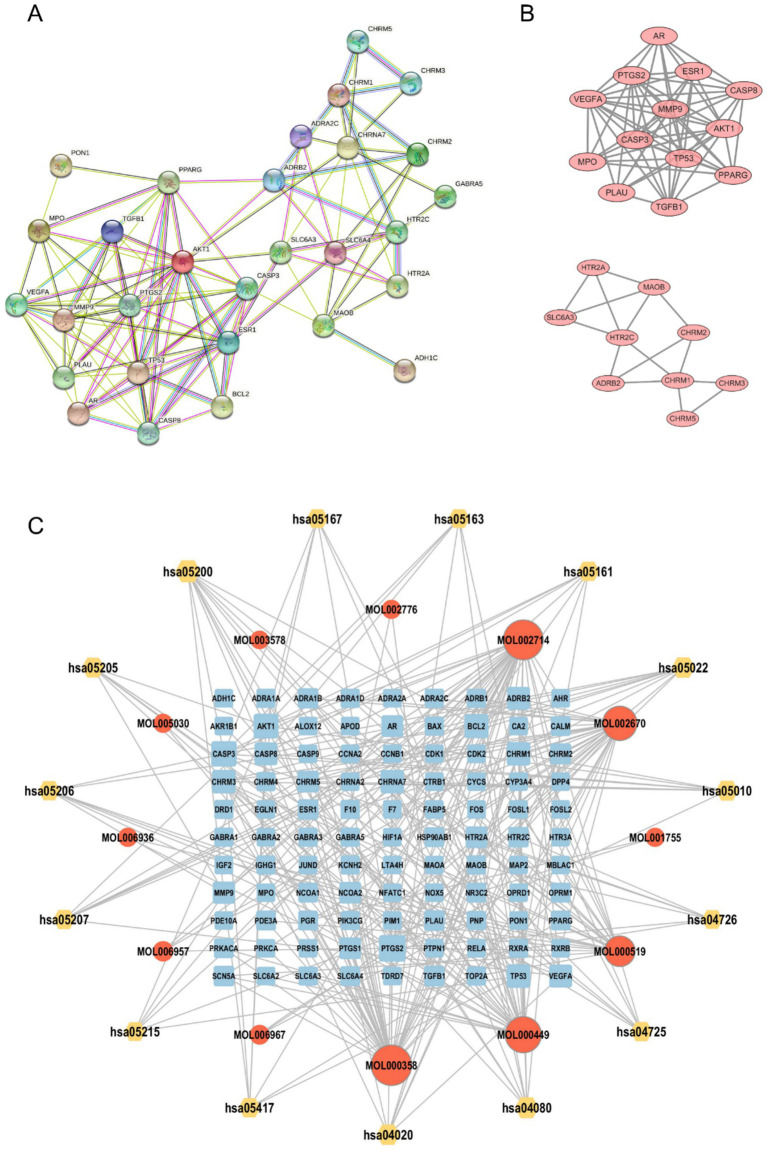
Comprehensive analysis of target genes. **(A)** The PPI network of the 29 target genes was constructed using the STRING database. **(B)** Two modules analyzed by Cytoscape. **(C)** Network showing the interactions between *Pinellia ternata* (Thunb.) Breit. components and target genes.

The PPI network of the 29 shared targets was constructed using the STRING database (Figure 2A), comprising 29 nodes and 112 edges. The network was further divided into two densely connected modules (Figure 2B), where proteins within the same module exhibited more frequent interactions. An integrated network of active components of *Pinellia ternata* (Thunb.) Breit., AD targets, and enriched pathways was visualized using Cytoscape v3.9.1 software (Figure 2C). In the network diagram, blue squares represent targets, red circles represent active components of *Pinellia ternata* (Thunb.) Breit., and yellow diamonds represent enriched pathways. Node size corresponds to the degree value, with larger nodes indicating greater importance within the network. Topological parameters were analyzed using NetworkAnalyzer to identify core AD targets and key active components mediating the therapeutic effects (Tables 3, 4). NetworkAnalyzer predicted that *β*-sitosterol, baicalein, stigmasterol, carotene, and coniferin are important active components of *Pinellia ternata* (Thunb.) Breit. for AD treatment (Table 3). Among these, β-sitosterol was identified as the most significant component, with a degree of 38, betweenness centrality of 0.2814, and closeness centrality of 0.4480. Baicalein ranked second, exhibiting a degree of 37, betweenness centrality of 0.3432, and closeness centrality of 0.4385. NetworkAnalyzer further identified PTGS2 as the primary target of *Pinellia ternata* (Thunb.) Breit. in AD treatment, with a degree of 14, betweenness centrality of 0.1402, and closeness centrality of 0.4980. CASP3 (degree: 12, betweenness centrality: 0.039845, closeness centrality: 0.399361) and AKT1 (degree: 11, betweenness centrality: 0.018782, closeness centrality: 0.342466) were also identified as key targets. Additional important targets implicated in the therapeutic effects of *Pinellia ternata* (Thunb.) Breit. on AD included BCL2, TP53, CHRM3, CHRM1, and CHRNA7 (Table 4).

**Table 3:** Network topological parameters of major active components of *Pinellia ternata* (Thunb.) Breit.

MOLOD	Molecule name	Degree	Betweenness centrality	Closeness centrality
MOL000358	beta-sitosterol	38	0.281492	0.448029
MOL002714	baicalein	37	0.343251	0.438596
MOL000449	Stigmasterol	31	0.23224	0.423729
MOL002670	Cavidine	28	0.202113	0.409836
MOL000519	coniferin	22	0.133703	0.396825

**Table 4:** Network topological parameters of key targets.

Target gene	Degree	Betweenness centrality	Closeness centrality
PTGS2	14	0.140268	0.498008
CASP3	12	0.039845	0.399361
AKT1	11	0.018782	0.342466
BCL2	10	0.032933	0.389408
TP53	9	0.009995	0.326371
CHRM3	9	0.02852	0.407166
CHRM1	9	0.02852	0.407166
CHRNA7	9	0.022152	0.384615
ADRB2	8	0.030219	0.404531
VEGFA	8	0.013777	0.340599

### 3.4 Molecular docking and molecular dynamics simulation

**Figure 3:**
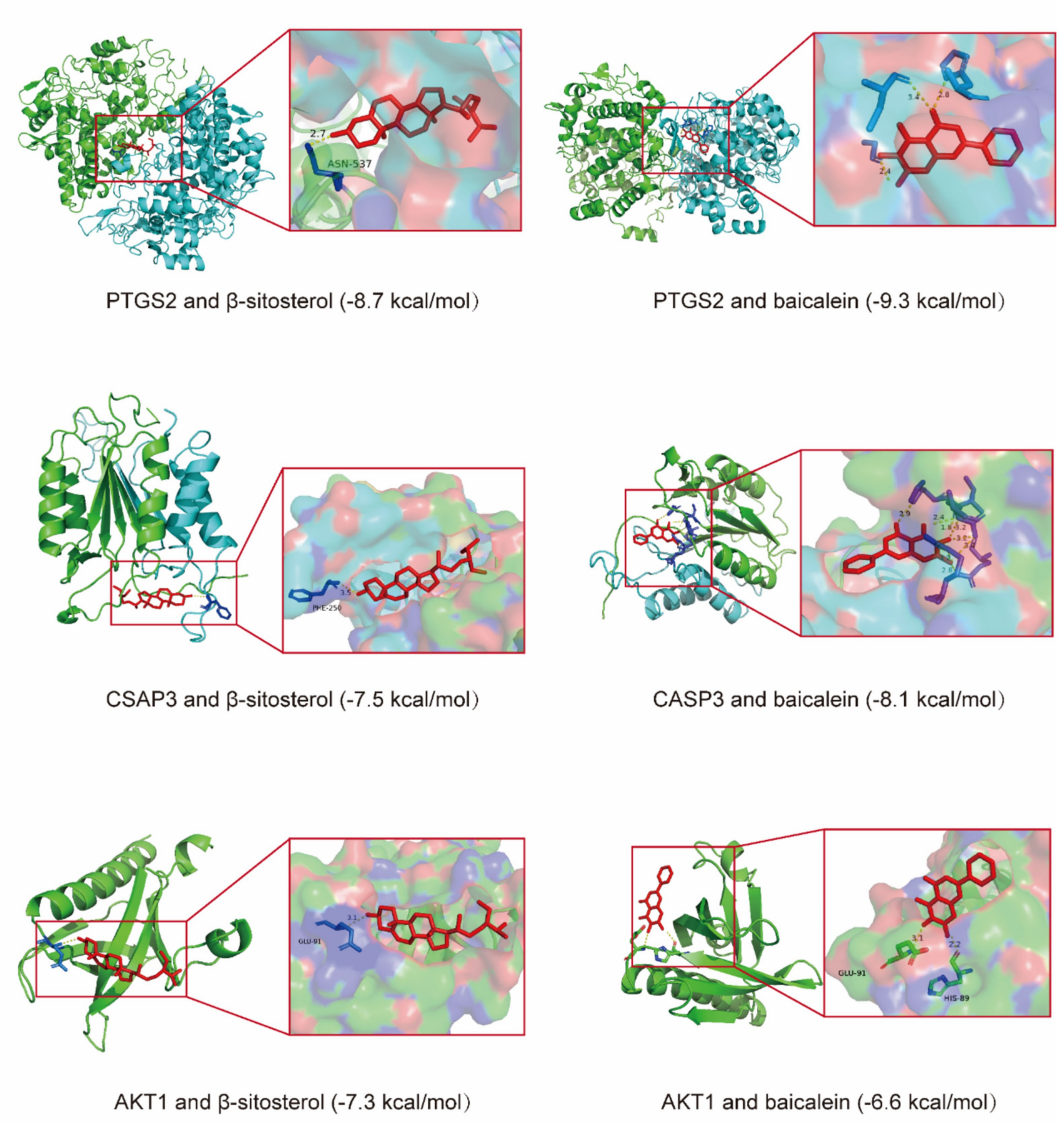
Molecular docking results of key active phytochemicals with active targets.

Three core targets with degrees greater than 10 and two key compounds identified in Section 2.3 were selected for molecular docking validation. The results showed that each candidate compound established visible hydrogen bonds with its corresponding protein target (Figure 3). Binding energies for all compound-target interactions were lower than −6 kcal/mol, indicating that the active components of *Pinellia ternata* (Thunb.) Breit. can stably associate with AD-related targets.

**Figure 4:**
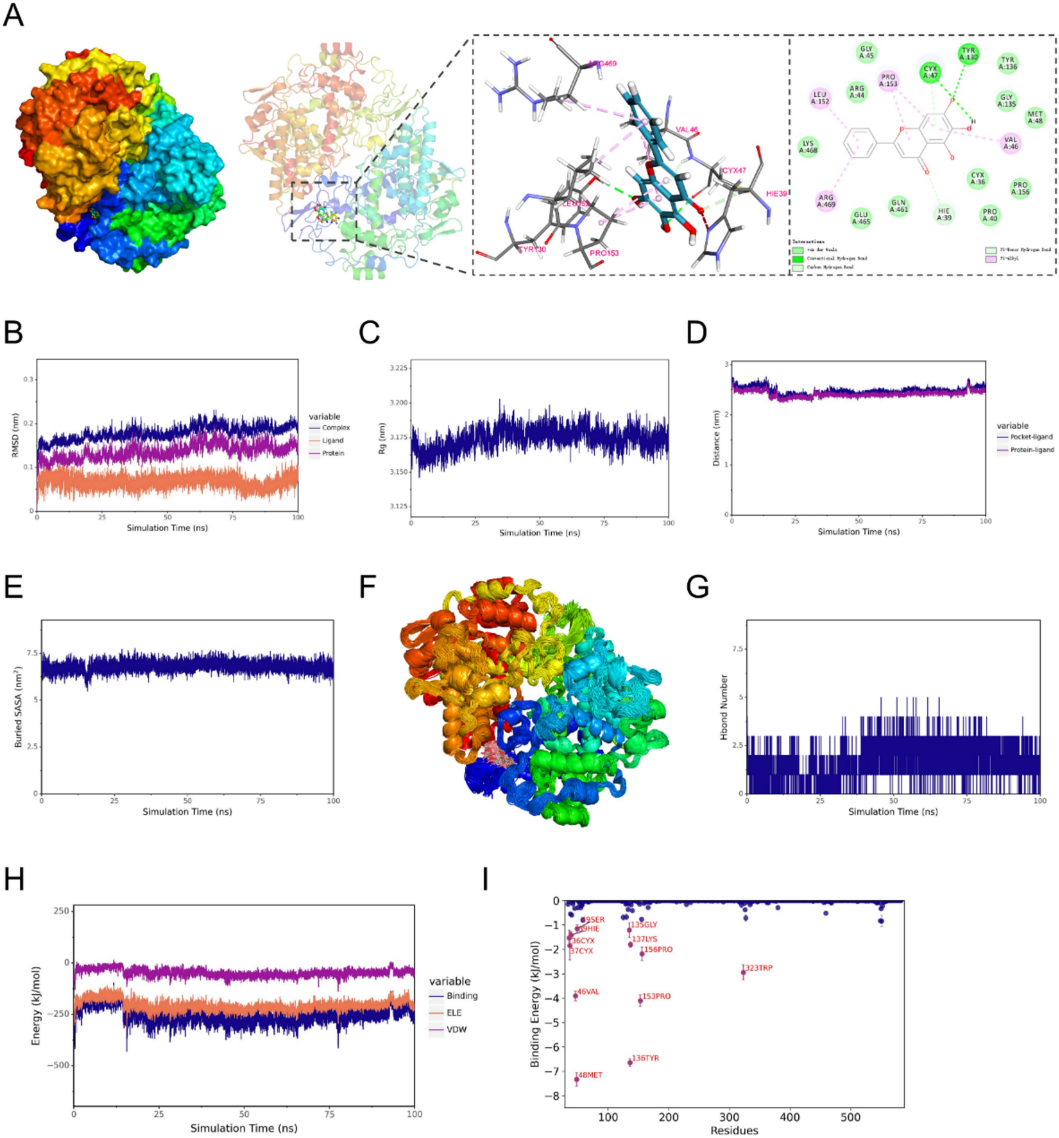
Molecular dynamics simulation of the PTGS2-baicalein. **(A)** 3D structure of protein-ligand binding mode, including binding pocket interaction diagram and 2D interaction plot. **(B)** RMSD curves of protein, ligand and complex. **(C)** Rg variation curve of protein complex. **(D)** Distance change between protein pocket and ligand. **(E)** Buried SASA curve of the complex in simulation. **(F)** Surface structure of target protein. **(G)** Hydrogen bond number variation between protein and ligand over simulation time. **(H)** Binding energy curves along simulation. **(I)** Per-residue decomposition of binding free energy.

The complex exhibiting the lowest binding energy, PTGS2-baicalein, was selected for MD simulation. As shown in Figure 4A, baicalein formed hydrogen bonds with residues TYR-130 and CYS-47 of PTGS2. Hydrophobic Pi-Alkyl interactions were observed with ARG-469, LEU-152, PRO-153, and VAL-46, while van der Waals interactions involved residues including GLU-465, PRO-156, and GLY-45.

MD simulation was subsequently conducted to further assess binding stability and characterize protein-ligand interactions. As shown in Figures 4B,C, the root-mean-square deviation (RMSD) of the complex gradually stabilized over the simulation time, and the radius of gyration (Rg) also converged, indicating structural equilibration. Analysis of center-of-mass distance (Figure 4D) revealed that the distance between the ligand and the protein binding site reached a stable state, confirming stable binding. The buried solvent-accessible surface area (Buried SASA) also achieved equilibrium (Figure 4E), indicating consistent ligand-protein contact throughout the simulation. Structural alignment of simulation snapshots (Figure 4F) revealed high conformational overlap of the ligand, indicative of its sustained occupation of the protein pocket. Hydrogen bonding, reflecting electrostatic interactions, fluctuated between 0 and 3 bonds during the simulation (Figure 4G). As shown in Figure 4H, van der Waals, hydrophobic, and electrostatic interaction energies gradually stabilized, supporting the formation of a stable complex. Binding free energy components were calculated using the Molecular Mechanics Poisson-Boltzmann Surface Area (MM-PBSA) method on equilibrated trajectory frames. The electrostatic interaction energy (ΔEele) was −12.576 ± 0.445 kcal/mol, van der Waals energy (ΔEvdw) was −155.395 ± 2.464 kcal/mol, polar solvation energy (ΔEpol) was 98.401 ± 1.848 kcal/mol, and nonpolar solvation energy (ΔEnonpol) was −17.87 ± 0.137 kcal/mol. The total binding free energy (ΔEMMPBSA) was −87.441 ± 1.526 kcal/mol. These results indicate that van der Waals interactions (ΔEvdw) play a dominant role in binding, while electrostatic (ΔEele) and hydrophobic (ΔEnonpol) interactions contribute to a lesser extent. The high negative binding energy reflects strong affinity between baicalein and PTGS2. Binding energy decomposition was performed to identify key residues contributing to the interaction. Residues with significant contributions, including MET-48 and TYR-136, are highlighted in Figure 4I, indicating their critical role in baicalein binding to PTGS2.

**Figure 5:**
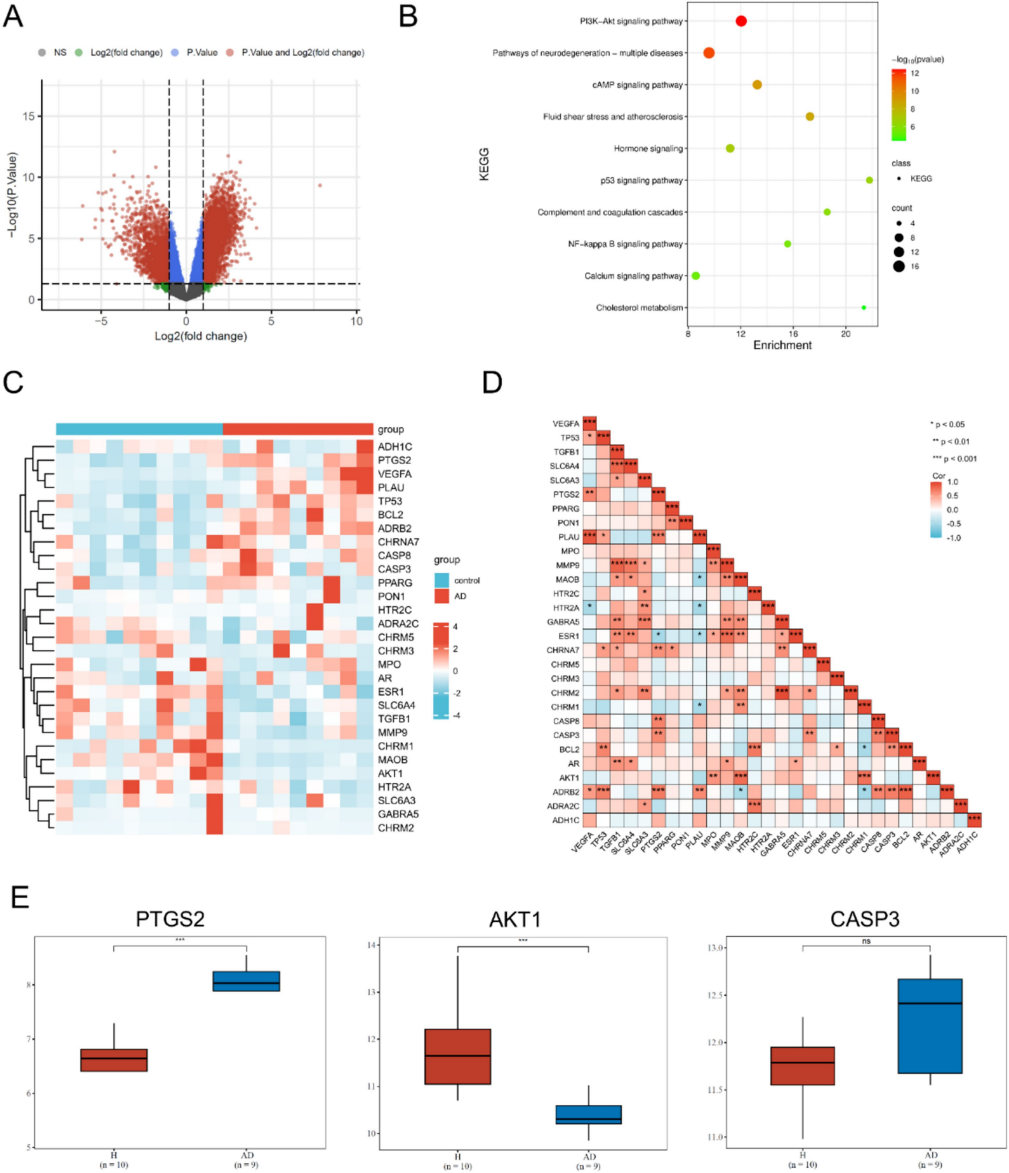
Differentially expressed genes in Alzheimer’s disease based on the GSE97760. **(A)** Volcano plot showing differential gene analysis in the GSE97760. **(B)** KEGG analysis of differential genes. **(C)** Heatmap exhibiting the expression levels of differential genes in AD and control samples. **(D)** The relationships among the differential genes evaluated by *p*-value. **(E)** External dataset validation in GSE97760 of PTGS2, AKT1 and CASP3.

### 3.5 Bioinformatics analysis

The gene expression profile series (GSE97760) was analyzed using GEO2R, yielding a total of 4,905 DEGs, including 2,922 upregulated and 1,983 downregulated genes. The intersection of these DEGs with AD-related genes from the database resulted in the identification of 111 DEGs, comprising 57 upregulated and 57 downregulated genes. KEGG pathway enrichment analysis of these DEGs revealed significant enrichment in pathways such as PI3K-Akt signaling, Pathways of neurodegeneration-multiple diseases, and cAMP signaling pathway. Comparison of the three core targets predicted in this study with the dataset showed elevated expression levels of PTGS2 and AKT1, whereas CASP3 expression showed no significant difference (Figure 5).

### 3.6 Effects of baicalein on Aβ1-42 induced injury in BV2 cells

**Figure 6:**
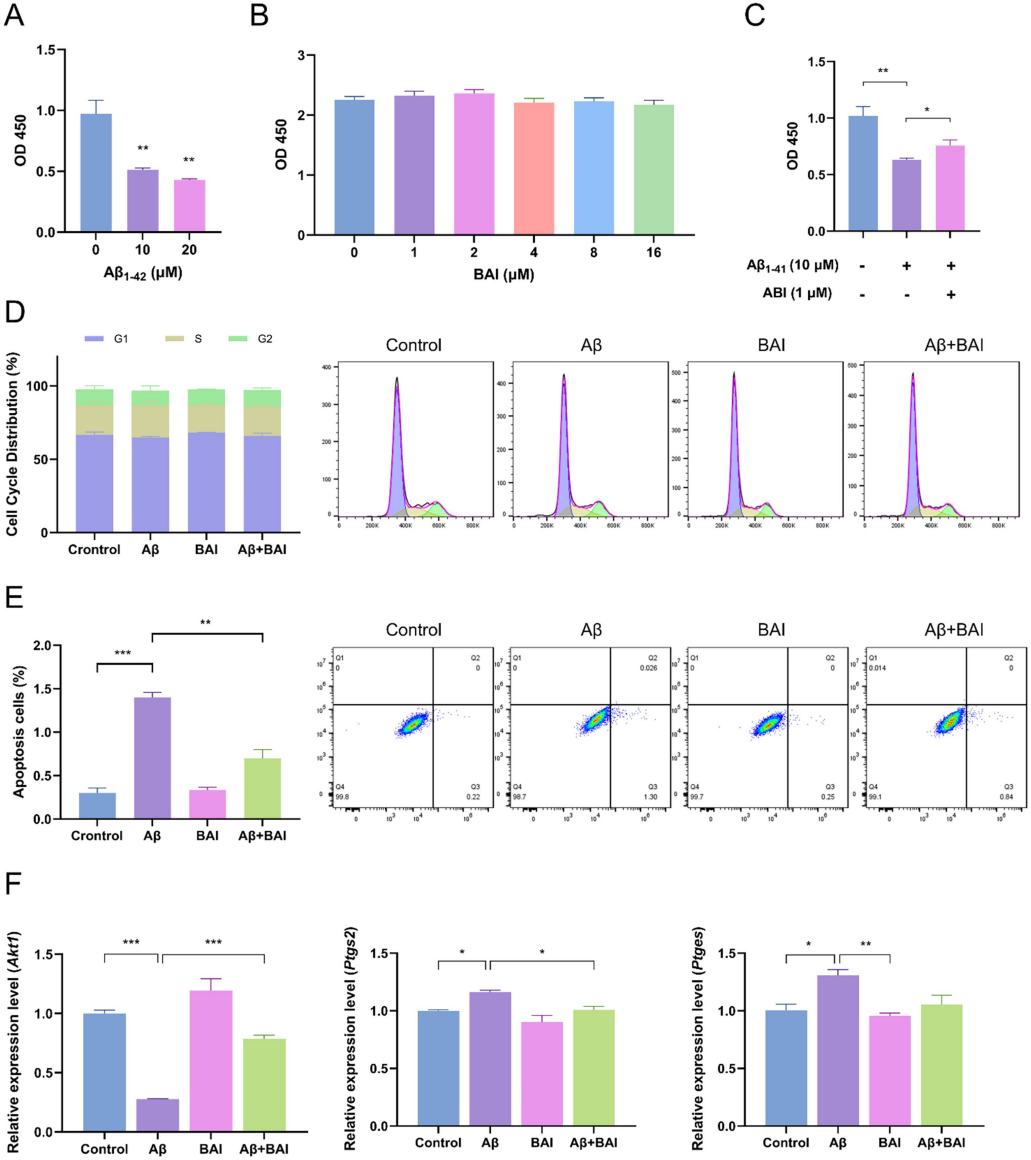
Experimental analysis of baicalein effects on BV2 cells. **(A)** Cytotoxic effect of Aβ1-42 on BV2 cells. **(B)** Cell activity of BV2 cells treated with different doses of baicalein. **(C)** BV2 cells were pretreated with baicalein, followed by a 24-h incubation with Aβ1-42. **(D)** The cell cycle of the BV2 cells was detected by flow cytometry. **(E)** The apoptosis of the BV2 cells was detected by flow cytometry. **(F)** mRNA expression of AKT1, PTGS2 and PGES in BV2 cells.

An Aβ_1-42_-induced injury model in BV2 cells was established using concentrations of 10 μM and 20 μM (Zhang et al., 2023a). CCK-8 assay results demonstrated that treatment with 10 μM Aβ_1-42_ for 24 h significantly reduced cell viability (*p* < 0.01; Figure 6A). Exposure of BV2 cells to varying concentrations of baicalein alone did not result in significant changes in cell viability (Figure 6B). Therefore, 1 μM baicalein was selected for subsequent experiments. Based on these results, BV2 cells were pretreated with 1 μM baicalein for 2 h, followed by co-incubation with Aβ_1-42_ for 24 h. CCK-8 assay demonstrated that Aβ_1-42_ treatment significantly inhibited BV2 cell proliferation, while baicalein pretreatment alleviated this inhibitory effect (Figure 6C).

### 3.7 Effects of baicalein on cell cycle and apoptosis in BV2 cells

To assess whether baicalein could mitigate Aβ_1-42_-induced perturbations in the cell cycle, DNA content distribution was analyzed by flow cytometry across different treatment groups. No significant changes in the cell cycle were observed in either the Aβ_1-42_-treated model group or the baicalein-pretreated group compared with the control (Figure 6D). These results indicate that Aβ_1-42_ did not significantly disturb the BV2 cell cycle, and baicalein alone had no significant effect on it.

Cell death was further evaluated by apoptosis analysis using Annexin V-FITC/PI double staining and flow cytometry to distinguish live, early apoptotic, and late apoptotic/necrotic cells. Aβ_1-42_ treatment significantly induced apoptosis in BV2 cells, particularly increasing in the proportion of early apoptotic cells. Baicalein pretreatment significantly reduced Aβ_1-42_-induced apoptosis (Figure 6E), demonstrating that baicalein exerts a protective effect against Aβ_1-42_-induced apoptosis in BV2 cells.

To investigate the regulatory effects of Aβ_1-42_ and baicalein on transcriptional levels of relevant genes, relative expression levels of AKT1 and PTGS2 were measured by qPCR (Figure 6F). Compared with the control group, AKT1 expression was significantly downregulated in the Aβ_1-42_ treatment group, whereas PTGS2 expression was significantly upregulated, consistent with the analysis of the GSE97760 dataset. In the baicalein-alone treatment group, AKT1 expression was significantly increased. In the baicalein pre-protection group, baicalein effectively reversed the Aβ_1-42_-induced downregulation of AKT1, and mitigated the upregulation of PTGS2, suggesting that baicalein can correct the abnormal gene expression of AKT1 and PTGS2 induced by Aβ_1-42_. To further validate the anti-neuroinflammatory mechanism of baicalein via PTGS2, mRNA levels of downstream inflammatory mediators were measured. Aβ_1-42_ treatment significantly upregulated PGE2, NFKB, TNFα and IL6 mRNA expression compared with the control group (Figure 6F, Supplementary Figure 1). Baicalein pretreatment markedly reduced the expression of pro-inflammatory genes. These results indicate that baicalein suppresses Aβ_1-42_–induced neuroinflammation at least in part by inhibiting the PTGS2/PGE2/NF-κB signaling axis.

## 4 Discussion

AD, the most prevalent neurodegenerative disorder, is characterized by irreversible progression and a high mortality rate (Scheltens et al., 2021). The precise pathogenesis of AD remains incompletely understood, with major hypotheses including the amyloid-beta cascade hypothesis, tau hyperphosphorylation hypothesis, central cholinergic impairment hypothesis, neuroinflammation hypothesis, and oxidative stress hypothesis. Current clinical pharmacotherapy primarily involves cholinesterase inhibitors, free radical scavengers, anti-amyloid agents, and anti-inflammatory drugs, none of which provide a cure. AD leads to severe consequences, including loss of self-care ability, memory impairment, and cognitive decline, imposing substantial psychological and economic burdens on patients and their families (Scheltens et al., 2021). With the global population ageing at an accelerated rate, AD has emerged as a major challenge in geriatric medicine (Hodson, 2018).

TCM, guided by a holistic perspective and the principles of syndrome differentiation-based treatment, has demonstrated unique advantages in the management of chronic complex diseases. Evidence suggests that TCM possesses distinctive strengths in the treatment of neurological disorders. Herbal medicines, owing to their multiple bioactive components, can act on multiple tissues and targets via various pathways to protect neurons and alleviate symptoms (Zhang et al., 2023b). *Pinellia ternata* (Thunb.) Breit. is a common component of TCM formulations used in neurological contexts, such as Ditan Decoction and Wendan Decoction. It exhibits documented pharmacological effects including anti-inflammatory, antitumor, and antioxidant activities. However, its specific pharmacodynamic material basis, core targets, and molecular regulatory network in AD remain poorly defined, limiting its clinical translation and development.

This study systematically elucidated the mechanism of *Pinellia ternata* (Thunb.) Breit. in the treatment of AD through an integrative approach combining network pharmacology, molecular simulation, and cellular experiments. The research identified baicalein and *β*-sitosterol as the core active components contributing to the anti-AD effects of *Pinellia ternata* (Thunb.) Breit., with PTGS2, CASP3, and AKT1 as the key targets. The interactions between these components and targets were predominantly enriched in pathways such as neuroactive ligand-receptor interaction, calcium signaling, and neurodegenerative disease pathways, reflecting the synergistic “multi-component, multi-target, multi-pathway” therapeutic characteristics of TCM. Baicalein (5,6,7-trihydroxyflavone) is a natural flavonoid compound with a typical flavone backbone structure. It is widely distributed in plants such as *Scutellaria baicalensis* Georgi and *Pinellia ternata* (Thunb.) Breit. Baicalein has been demonstrated to upregulate neuroprotective proteins and ameliorate cognitive impairment (Kuwar and Singh, 2025). Additionally, studies have demonstrated its potential neuroprotective effects in Parkinson’s disease models by inhibiting oxidative stress and alleviating MPP^+^/MPTP-induced neurotoxicity (Song et al., 2021). Moreover, baicalein has been proven to have a direct antagonistic effect on Aβ neurotoxicity in neuronal models, reducing neuronal apoptosis and improving synaptic function (Lin et al., 2017; Gao et al., 2020). β-Sitosterol, a phytosterol widely present in plants such as *Polygonum multiflorum* and *Houttuynia cordata*, exhibits antioxidant, anti-inflammatory, anti-proliferative, and anti-tumor properties and has been used in the treatment of various diseases (Nandi et al., 2024). Dietary β-sitosterol can penetrate the brain and accumulate in the plasma membranes of neural cells, where it activates the PI3K-GSK3β signaling pathway, thereby inhibiting oxidative stress and lipid peroxidation induced by glucose oxidase (Shi et al., 2013).

The active components of the *Pinellia ternata* (Thunb.) Breit. share 29 overlapping target genes with known AD-related targets. KEGG pathway enrichment analysis of these intersecting genes indicates that *Pinellia ternata* (Thunb.) Breit. may exert therapeutic effects in AD through multiple pathways, primarily involving Pathways in cancer, Neuroactive ligand-receptor interaction, Calcium signaling pathway, and Pathways of neurodegeneration. The neuroactive ligand-receptor interaction pathway is directly associated with neural function, and its disruption can result in memory impairment. Amyloid deposition in AD enhances ryanodine receptor-mediated calcium influx, resulting in dysregulation of calcium signaling and subsequent neuronal death. The pathways of neurodegeneration encompass mechanisms such as abnormal protein dynamics due to autophagy deficiency, oxidative stress, and free radical formation. Key targets of *Pinellia ternata* (Thunb.) Breit., including CASP3, CHRM5, BCL2, and PTGS2, participate throughout these processes and play significant roles in the progression of neurodegenerative diseases.

PPI network analysis revealed that the active components of *Pinellia ternata* (Thunb.) Breit. primarily act on targets such as PTGS2, CASP3, AKT1, BCL2, TP53, and CHRM3, all of which are closely associated with neurological disorders. Previous studies have shown that PTGS2 expression is significantly elevated in mice with cerebral ischemia/reperfusion injury, and treatment with arbutin can downregulate PTGS2 to ameliorate pathological outcomes. Upregulated PTGS2 has also been observed in neuronal AD models treated with Aβ_1-42_, where miR-125b inhibits PTGS2 to promote neuronal growth and suppress apoptosis and inflammation (Zhuang et al., 2020). Consistently, the present study found that PTGS2 is highly expressed in Aβ_1-42_-induced AD model neurons, and baicalein effectively inhibits this pathological process. Additionally, elevated PTGS2 expression was confirmed in blood samples from patients with AD in the GEO database, supporting its clinical relevance. We further detected downstream indicators of the PTGS2 pathway. Aβ_1-42_ markedly increased PGE2 mRNA expression, which was significantly reversed by baicalein pretreatment. CASP3, a cysteine-aspartic protease, is central to the execution phase of apoptosis and serves as a key enzyme in amyloid precursor protein cleavage, thereby contributing to neuronal apoptosis in AD. CASP3 is also capable of inducing abnormal tau phosphorylation, leading to neurofibrillary tangle formation and cognitive deficits (Olivera Santa-Catalina et al., 2017). AKT1, a neuroprotective protein, regulates various biological processes including cellular metabolism, proliferation, division, and angiogenesis, and is crucial for normal nervous system development and memory formation. Evidence suggests that the AKT1 rs2498786 gene polymorphism may be associated with AD susceptibility (Liu et al., 2015). Downregulation of AKT1 in AD models impairs neuronal survival signaling, while activation of AKT1 by baicalein may provide critical neuroprotective effects. Although this study did not detect the phosphorylation level of AKT protein and the activity of PI3K/Akt pathway, previous studies have shown that baicalein can indeed activate the PI3K/Akt signaling pathway in other neurological disease models. In the model of cerebral ischemia–reperfusion injury, baicalein can alleviate neuronal apoptosis by upregulating p-AKT levels (Yang et al., 2019; Li et al., 2020). In Parkinson’s disease models, baicalein has also been shown to activate the PI3K/Akt pathway and exert neuroprotective effects (Zhang et al., 2012). Therefore, we speculate that baicalein may also inhibit apoptosis in BV2 cells induced by A*β*_1-42_ by regulating the PI3K/Akt signaling pathway.

To evaluate the binding energy and interaction patterns between the small-molecule active components of *Pinellia ternata* (Thunb.) Breit. and the predicted targets, molecular docking and MD simulations were performed. The binding energy between baicalein and PTGS2 was determined to be as low as −9.3 kcal/mol. Stable binding was primarily mediated by van der Waals interactions, with key residues MET-48 and TYR-136 contributing substantially to the interaction energy, confirming the specificity of this binding and providing atomic-level evidence supporting the mechanism by which baicalein targets PTGS2.

Furthermore, we use BV2 to construct a neural injury model. Microglia are key immune cells in the central nervous system and play a central role in the neuroinflammatory response of AD (Fazal et al., 2026). Previous literature has shown that the Aβ-induced BV2 cell inflammation model has been widely used for the initial screening of anti-inflammatory and neuroprotective drugs (Cai et al., 2017; Ding et al., 2020). Cell-based experiments demonstrated that in an Aβ_1-42_-induced BV2 microglial cell injury model, pretreatment with 1 μM baicalein significantly alleviated the suppression of cell proliferation and exerted neuroprotective effects by inhibiting early apoptosis. Notably, baicalein did not produce significant alterations in the cell cycle of BV2 cells, suggesting that its neuroprotective activity primarily relies on anti-apoptotic mechanisms rather than modulation of cell proliferation. This observation provides a clear rationale for subsequent mechanistic studies.

This study has several limitations. First, it focused exclusively on the validation of a single component, baicalein, without examining potential synergistic effects with other bioactive constituents, such as β-sitosterol and stigmasterol. Future studies should evaluate the combined effects of multiple components to better reflect the holistic characteristics of traditional Chinese medicine. Second, the cellular experiments were conducted solely in BV2 microglial cells, without validation in neuronal (e.g., SH-SY5Y) or astrocytic models, thereby limiting the ability to fully recapitulate the neural microenvironment *in vivo*. Furthermore, the regulatory role of PTGS2 on downstream inflammatory mediators remains to be elucidated, and the molecular mechanisms underlying the core signaling pathways require further investigation. Future studies could employ multicellular co-culture models, APP/PS1 double-transgenic mouse models, and techniques such as Western blot, immunofluorescence, and behavioral assays (e.g., Morris water maze) to validate the synergistic effects of multiple active components of *Pinellia ternata* (Thunb.) Breit. and the *in vivo* efficacy of the “baicalein-PTGS2-neuroinflammation” axis. The calcium signaling and neuroactive ligand-receptor interaction pathways remain to be verified in future studies to fully illustrate the multi-target mechanism of *Pinellia ternata* (Thunb.) Breit.

## 5 Conclusion

In conclusion, this multidisciplinary study elucidated the core active components, key targets, and molecular pathways underlying the therapeutic effects of *Pinellia ternata* (Thunb.) Breit. in AD. Baicalein, as one of the key active components, protects BV2 cells from Aβ_1-42_-induced injury by -targeting PTGS2 and modulating neuroinflammatory pathways. These findings not only provide a candidate compound and a potential molecular target for the development of novel AD therapeutics derived from TCM but also establish a theoretical framework for the clinical application and further investigation of *Pinellia ternata* (Thunb.) Breit.

## Data Availability

The original contributions presented in the study are included in the article/Supplementary material, further inquiries can be directed to the corresponding authors.
