# Supplementary material for: Protective effect of baicalein from Pinellia ternate on Alzheimer’s disease cell injury: a network pharmacology, molecular docking, and molecular dynamics study
Source: Front Aging Neurosci. 2026 Jun 11;18:1848282. doi: 10.3389/fnagi.2026.1848282 (PMC13294263; doi:10.3389/fnagi.2026.1848282)
Supplement: Supplementary file 1 [file Table_1.DOCX]

Supplementary Material

# Supplementary Figure

**
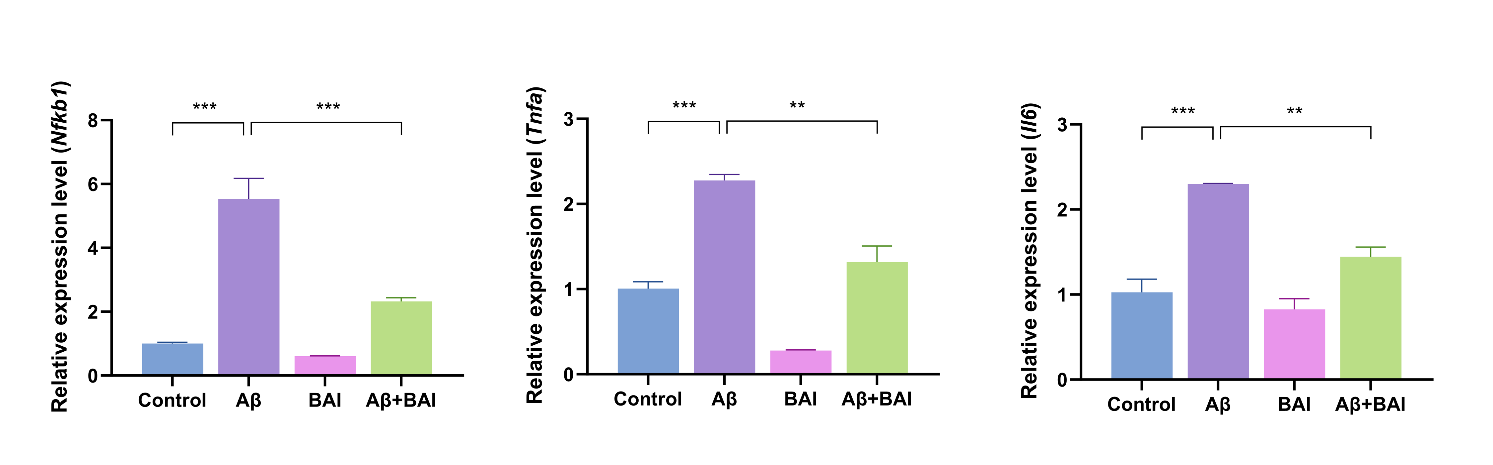
**

**Supplementary Figure 1.** mRNA expression of NFκB, TNFα and IL6 in BV2 cells.
